# Know-do gaps in the clinical management of childhood illness: evidence from three countries in sub-Saharan Africa

**DOI:** 10.1186/s12889-025-24852-0

**Published:** 2025-10-24

**Authors:** Emma Clarke-Deelder, Pablo Amor Fernandez, Salome Drouard, Eeshani Kandpal, Günther Fink, Gil Shapira

**Affiliations:** 1 Swiss Tropical & Public Health Institute, Kreuzstrasse 2, Allschwil, 4123 Switzerland; 2 University of Basel, Basel, Switzerland; 3 The World Bank, Washington, DC USA; 4 University of Washington, Seattle, USA; 5 Center for Global Development, Washington, DC USA

**Keywords:** Child health, Sub-Saharan africa, Quality of care, Malaria, Pneumonia, Dehydration

## Abstract

**Background:**

After neonatal conditions, the leading causes of child mortality in sub-Saharan Africa are malaria, lower respiratory infections, and dehydration. Many of these deaths could be averted with basic and widely-available health interventions, but quality of care remains low. We aimed to assess adherence to clinical guidelines for these conditions in Burundi, the Democratic Republic of the Congo (DRC), and Nigeria, and estimate the proportion of guideline non-adherence that is explained by gaps in health care provider knowledge versus other factors.

**Methods:**

We conducted an observational study in randomly-sampled health facilities in each study country, linking data from direct observations of under-5 sick child visits, knowledge assessments of the treating health care providers, and interviews with caregivers. For children diagnosed with malaria, severe respiratory infection, or dehydration, we defined the “adherence gap” as the percentage who did not receive correct treatment, and the “know-do gap” as the percentage who received incorrect care despite the provider knowing the correct treatment. We evaluated the portions of overall adherence gaps that were explained by know-do gaps, and described factors associated with know-do gaps.

**Results:**

A total of 2,212 sick child visits treated by 852 providers were analyzed. In the pooled sample, 87%, 75%, and 77% percent of providers were familiar with the main treatment recommendations for malaria, pneumonia, and dehydration, respectively. When observed by survey staff during consultations with sick children, compliance with the same guidelines was 76%, 74%, and 51%. Knowledge gaps explained between 0% of the total adherence gap for pneumonia treatment in Burundi and 40% of the gap for pneumonia treatment in the DRC.

**Conclusions:**

To improve quality of care, it is critical to understand why providers do not consistently follow clinical guidelines. Our findings suggest that adherence to protocols is low, but that knowledge is not the primary barrier. Interventions to improve quality must go beyond improving knowledge to also address other drivers of provider behavior such as motivation, workload, and systemic constraints.

**Supplementary Information:**

The online version contains supplementary material available at 10.1186/s12889-025-24852-0.

## Background

 Child mortality in sub-Saharan Africa remains a critically important challenge: 7% of children born in the region die before they reach age five [1]. More than one third (39%) of these deaths occur in the first 28 days of life from neonatal disorders such as congenital birth defects and complications from preterm birth [2]. Excluding neonatal disorders, the leading causes of under-five mortality in sub-Saharan Africa are lower respiratory infections (20%), diarrhea (20%), and malaria (19%) [2]. Many deaths from these conditions are preventable with simple and widely available interventions such as antibiotics, antimalarial drugs, and oral rehydration solution (ORS). However, these interventions are not consistently delivered to the children who need them in these settings [3–7].

Improving the technical quality of care – defined as the correct use of evidence-based clinical practices – is critical to reducing child mortality. Despite significant investments to strengthen health systems in low- and middle-income countries, many health facilities continue to deliver suboptimal technical quality of care [8, 9]. This gap between access and quality care undermines progress towards achieving the Sustainable Development Goals (SDGs), which emphasize not only health care availability but also quality and impact [10, 11].

One potential explanation for low technical quality of care is insufficient training on clinical guidelines. However, even with appropriate knowledge, providers may not always follow recommended guidelines in practice. This disconnect between what health workers know and what they do in practice—referred to as the know-do gap—has been documented across a range of settings and clinical areas including primary care in Tanzania [12], maternity care in Uganda [13], tuberculosis and HIV care in South Africa and India [14, 15], and child health care in India, Ethiopia, and Liberia [16–19]. These findings underscore the importance of measuring and understanding the know-do gap across diverse clinical and geographic contexts. Doing so can inform more targeted quality improvement strategies by clarifying whether poor care quality is primarily a result of knowledge deficits or other systemic or behavioral barriers. Where know-do gaps are large, it suggests that improving provider knowledge alone may be insufficient, and that broader structural or motivational constraints must also be addressed.

To better understand the relative importance of knowledge versus know-do gaps in child health care, we conducted a multi-country study of knowledge of and adherence to clinical guidelines for management of common childhood illnesses. Using a new and rich dataset containing direct observations, health care provider interviews, and caregiver interviews in Burundi, the Democratic Republic of the Congo (DRC), and Nigeria, we assessed health care providers’ knowledge of clinical protocols and measured the technical quality of curative care given by the same health care providers to children in health facilities. Our focus was on the main causes of child mortality after the neonatal period. By evaluating the gaps between provider knowledge and clinical practice, our study aims to inform targeted quality improvement strategies in these high-burden settings.

## Methods

### Study design

We conducted a cross-sectional study in random samples of health facilities in two low-income countries (Burundi and the DRC) and one middle-income country (Nigeria). Data collection took place in 2013 (Nigeria), 2014 (Burundi), and 2015 (DRC). The data were collected as part of the baseline surveys for randomized controlled trials of results-based financing (RBF) programs. These data were collected before the implementation of RBF, so our results are not affected by the interventions and adherence levels represent pre-intervention adherence.

### Setting

The study countries are highly diverse. At the time of data collection, Gross National Income per capita (Purchasing Power Parity-adjusted) ranged from USD 730 in Burundi to USD 4,880 in Nigeria. Under-5 mortality ranged from 72 deaths per 1,000 live births in Burundi to 130 deaths per 1,000 live births in Nigeria, and population size ranged from 10 million in Burundi to 175 million in Nigeria [1].

While these countries are diverse in many ways, they share the main causes of child mortality, and all have adopted the WHO’s Integrated Management of Childhood Illness (IMCI) strategy and guidelines. The IMCI strategy was introduced in the mid-1990s to provide a coherent evidence-based approach to care for childhood illnesses [20]. It includes three main components: “improving case management skills of health workers, improving health systems to provide quality of care, and improving family and community practices for health, growth, and development” [21].The IMCI guidelines provide algorithm-based recommendations for classifying children’s signs (such as pallor and rapid breathing) and main symptoms (such as cough, diarrhea, and fever) into severe and non-severe forms of childhood illnesses including pneumonia, malaria, and dehydration. The guidelines were designed to be implemented by non-physician health workers. The IMCI strategy was adopted in Nigeria in 1997, in the DRC in 1999, and in Burundi in 2003 [22, 23]. Recent reviews of global IMCI implementation have documented very high rates of national adoption, but numerous challenges in implementation, including insufficient training, mentorship, and supervision [9, 24].

In all three countries, primary care for children is provided through health centers at the community level. In Nigeria, the Ward Minimum Health Care Package provides a set of health interventions in primary health care centers, including routine child health care, for free or at low cost to patients [25]. Community Health Workers (CHWs) play a significant role in the provision of routine child health care, including implementation of the IMCI guidelines [26]. In the Democratic Republic of Congo (DRC), primary care for children is provided in health posts, health centers, and hospitals managed by the Ministry of Health, with health care provider salaries largely financed through user fees [27]. In Burundi, the organization of primary care for children is also mainly through health centers that are often staffed by nurses.

### Participants

Health facilities were randomly selected to participate in evaluations of World Bank-supported RBF programs in each country. The sample in Burundi was nationally representative, while the samples in the DRC and Nigeria were representative at the subnational (health zone and state) levels. The sample in Burundi included only health centers. The sample in the DRC included health centers, reference health centers (which provide a package of services similar to hospitals and typically have a doctor on staff), and referral hospitals. The sample in Nigeria included health posts (where community health workers deliver services), health centers, and hospitals. Sampling frames in each country were provided by national or subnational health authorities. Further details on the sampling approaches are included in evaluation reports from each study country [28–32].

Within health facilities, data collection included health facility assessments, health care provider interviews, observations of consultations with patients under 5 years of age, and interviews with caregivers of children under 5 years of age. Children receiving outpatient care were randomly sampled for participation. In our analysis, we excluded children under the age of 2 months because IMCI guidelines differ for this age group and are not primarily focused on the infectious diseases outlined above. We also excluded children who were in the health facility for a well-child visit (e.g., vaccination or growth monitoring).

Health care providers were eligible for participation if they routinely take care of children under 5 as part of their role.The health care provider sample included doctors (in Nigeria and the DRC), nurses, community health workers (in Nigeria), and other cadres of health workers such as health assistants (particularly in Burundi).

Since this study was a secondary analysis of data from the RBF evaluations, we did not complete sample size calculations. All participants meeting the inclusion criteria were included in the analysis.

### Data sources

In this study, we analyzed four types of data that were collected as part of facility-based surveys developed for evaluations of results-based financing interventions in each study country [33–35]:

#### Health care provider interviews

In each facility, up to five health care providers were randomly selected for an interview, which included questions about their background as well as vignettes to assess clinical knowledge. In vignettes, participants were asked to describe how they would treat imaginary patients. Vignettes were implemented differently in different countries. In Nigeria, the questions were open-ended: interviewers asked participants a question (e.g., “what treatment would you provide in this case?”) and then the respondent listed answers in response, without being prompted. In the DRC, the responses were prompted: the interviewer listed a set of possible treatments and the participant indicated which, among these, they would provide. Finally, in Burundi, the vignettes were dynamic. Instead of providing all information about the case upfront, interviewers only provided participants with certain pieces of information (e.g., the child’s temperature) if the participant asked about it. Vignettes are described in more detail in Tables S1-S3.

#### Direct observations of care

Data on adherence to clinical guidelines were collected through direct observations of the same health workers taking care of sick children at their health facility. Trained observers – typically medical doctors or nurses - used a structured checklist to carefully document the care provided during consultations. Observers recorded all diagnostic assessments conducted and questions asked by the provider.

#### Caregiver interviews

After consultations were observed, exit interviews were conducted with the caregivers of the sick children. During these interviews caregivers were asked to report on their child’s visit to the health facility, including any prescriptions or medications given (or purchased after the visit), costs, and the general quality of the consultation.

#### Health facility assessments

Finally, a health facility assessment was completed for each facility. This included information about the facility type, infrastructure, patient volume, and supply availability (in the DRC and Nigeria).

The questionnaires used in the DRC and Nigeria are available online [29, 33]; the questionnaires used in Burundi are available by request to the original trial study authors [35].

### Measurement and primary outcome variable

The primary outcome of interest was compliance with IMCI guidelines for treating the main causes of child mortality after the neonatal period. We focused on three key recommended and relatively straightforward actions: the use of antimalarials (including quinine and artemisinin-based antimalarials) for diagnosed malaria cases; the use of oral rehydration solutions (ORS) or IV fluids (saline or ringer’s lactate) for diagnosed dehydration cases; and the use of antibiotics for diagnosed pneumonia or other severe respiratory infections (including severe pneumonia, unspecified severe respiratory infection, and pertussis) requiring an antibiotic.

As a secondary analysis, we estimated adherence to stricter treatment recommendations: amoxicillin for pneumonia or other severe respiratory infection cases, and artemisinin-containing antimalarial medications for malaria cases. For dehydration cases, we also reported whether providers recommended that parents provide additional fluids to their child (though this is not considered sufficient treatment in the IMCI guidelines).

### Statistical methods

We began by describing the facilities, providers, and patients in the sample. We then described provider knowledge based on clinical vignettes. Third, we described the technical quality of care observed during sick child consultations. Finally, we reported know-do gaps, measuring, for each illness, the proportions of observed consultations in which providers [1] demonstrated knowledge of the relevant clinical protocols during their knowledge assessment and gave the recommended treatment in practice (“Know and do”) [2], gave the recommended treatment despite not demonstrating the relevant knowledge (“Don’t know and do”) [3] did not give the recommended treatment, despite demonstrating knowledge of the relevant clinical protocols (“Know and don’t do”), and [4] neither demonstrated knowledge of the protocol nor gave the recommended treatment (“Don’t know and don’t do”). The overall adherence gap includes cases of “know and don’t do” and cases of “don’t know and don’t do.” Cases of “know and don’t do” reflect a know-do gap, while cases of “don’t know and don’t do” reflect a knowledge gap.

To understand the extent to which know-do gaps might be explained by stockouts of needed medications, we also examined know-do gaps in the subset of facilities where we know (from facility assessments) that the relevant medications were available. We included this as a secondary analysis rather than the main analysis because providers can also prescribe medicine, regardless of whether it is practical for caregivers to access a pharmacy. In another secondary analysis, we assess whether know-do gaps are smaller for patients diagnosed with severe forms of pneumonia and dehydration (though not malaria, as severity of malaria diagnoses was not documented during data collection).

Finally, we analyzed factors associated with know-do gaps. We examined bivariate associations between facility and provider characteristics and know-do gaps, by country and condition. We then used multivariate linear regression models to assess the association between correct treatment and facility and provider characteristics, restricting our sample to cases in which providers were correct on the relevant treatment assessment(s). In this analysis, we pooled the three conditions; if children have multiple diagnoses, we coded them as correctly treated if they received the recommended treatment for all their diagnosed conditions. Coefficients from this analysis can be interpreted as the percentage point change in the adherence rate associated with a 1-unit change in each of the covariates. Missing data were assumed to be missing completely at random, meaning that missingness is independent of the observed and unobserved variables [36].

All statistical analyses were conducted using Stata version 13.

## Results

The analytic sample included 2,212 children across the three study countries (Table 1, Fig.**S1**). These children were seen by 852 health care providers in 723 unique health facilities.

**Table 1:** Facility, provider, and patient characteristics

	Burundi	DRC	Nigeria	Pooled
Facility characteristics	*N* = 89	*N* = 255	*N* = 379	*N* = 723
*Facility type*	*N (%)*	*N (%)*	*N (%)*	*N (%)*
Hospital	0 (0.0%)	41 (16.1%)	7 (1.8%)	48 (6.6%)
Reference health center	0 (0.0%)	33 (12.9%)	0 (0.0%)	33 (4.6%)
Other health center	89 (100.0%)	181 (71.0%)	356 (93.9%)	626 (86.6%)
Health post	0 (0.0%)	0 (0.0%)	16 (4.2%)	16 (2.2%)
*Other facility characteristics*				
	*Mean (SD)*	*Mean (SD)*	*Mean (SD)*	*Mean (SD)*
Under-5 patients in the past month (as recorded in the register)^1^	-	115 (146)	54 (176)	80 (166)
	*N (%)*	*N (%)*	*N (%)*	*N (%)*
Facility has a sink with soap and water^2^	-	103 (46.6%)	187 (59.6%)	290 (54.2%)
Facility has electricity^3^	-	163 (63.9%)	247 (65.2%)	410 (64.7%)
Facility owns a means of transportation^4^	-	34 (13.3%)	42 (11.1%)	76 (12.0%)
Provider characteristics	*N* = 144	*N* = 289	*N* = 419	*N* = 852
* Cadre* ^5^	*N (%)*	*N (%)*	*N (%)*	*N (%)*
Doctor	0 (0.0%)	26 (9.0%)	14 (3.4%)	40 (4.7%)
Nurse	94 (65.3%)	251 (86.9%)	54 (13.1%)	399 (47.2%)
Community Health Worker	0 (0.0%)	0 (0.0%)	312 (75.5%)	312 (36.9%)
Other^6^	50 (34.7%)	12 (4.2%)	33 (8.0%)	95 (11.2%)
*Other provider characteristics*				
	*Mean (SD)*	*Mean (SD)*	*Mean (SD)*	*Mean (SD)*
Age (years)^7^	33 (8)	40 (9)	43 (8)	40 (9)
	*N (%)*	*N (%)*	*N (%)*	*N (%)*
Female gender	68 (47.2%)	37 (12.8%)	251 (59.9%)	356 (41.8%)
Ever participated in IMCI training^8^	-	53 (18.4%)	164 (39.5%)	217 (30.9%)
Participated in IMCI training in the past 12 months^9^	-	15 (5.2%)	60 (14.5%)	75 (10.7%)
Patient characteristics	*N* = 501	*N* = 936	*N* = 775	*N* = 2,212
*Symptoms reported by the caregiver*	*N (%)*	*N (%)*	*N (%)*	*N (%)*
Fever	293 (58.5%)	760 (81.2%)	556 (71.7%)	1608 (72.7%)
Cough	123 (24.6%)	390 (41.7%)	314 (40.5%)	827 (37.4%)
Diarrhea	143 (28.5%)	185 (19.8%)	230 (29.7%)	557 (25.2%)
* Diagnosis*	*N (%)*	*N (%)*	*N (%)*	*N (%)*
Malaria	131 (26.1%)	670 (71.6%)	183 (23.6%)	984 (44.5%)
Dehydration	6 (1.2%)	72 (7.7%)	30 (3.9%)	108 (4.9%)
Pneumonia or other respiratory infection	20 (4.0%)	199 (21.3%)	55 (7.1%)	274 (12.4%)
*Other patient characteristics*				
Female gender^10^		430 (45.9%)	371 (47.9%)	801 (46.8%)
	*Mean (SD)*	*Mean (SD)*	*Mean (SD)*	*Mean (SD)*
Age (months)^11^	25 (14)	25 (15)	21 (15)	24 (15)

^1^Not available for Burundi. Missing data for 7 facilities in the DRC, and 40 facilities in Nigeria

^2^Not available for Burundi. Missing data for 34 facilities in the DRC, and 65 facilities in Nigeria

^3^Not available for Burundi.

^4^Not available for Burundi.

^5^Missing data for 5 providers in Nigeria.

^6^This category includes participants who chose ‘other’ in their response to the question about their cadre, as well as several cadres that had small numbers of participants such as “aide-soignant” (health assistant) and “chief of service"

^6^Missing data for 2 providers in Nigeria

^7^Missing data for 1 provider in DRC and 4 providers in Nigeria

^8^Missing data for 1 provider in DRC and 4 providers in Nigeria

^9^Not available in Burundi

^10^Missing data for 63 patients in Burundi

The majority of health facilities in the study sample were health centers. The cadres of providers varied across countries: most of the providers in the Burundi and DRC samples were nurses (65% and 87%), while most of the providers in the Nigeria sample were Community Health workers (76%). The rate of participation in formal in-service IMCI training was 18% in DRC and 40% in Nigeria (with no data in Burundi).

The most common reason that caregivers reported for the health facility visit was fever, followed by cough and then diarrhea. In the pooled sample, 984 children (45%) were diagnosed with malaria, 274 (12%) were diagnosed with pneumonia or another severe respiratory infection, and 108 (5%) were diagnosed with dehydration.

Knowledge of clinical protocols was generally high (Table 2). For instance, across countries, 87% of providers recommended giving a patient with malaria an antimalarial drug (including quinine). 75% of providers recommended any antibiotic for a patient with pneumonia or other severe respiratory infections. 77% of providers recommended fluids (ORS, saline, or ringer’s lactate) for patients with signs of dehydration. Results were similar when we restricted the sample for each knowledge assessment to the providers who were observed caring for patients with the relevant condition (Table S4).

**Table 2:** Provider knowledge of clinical protocols for the management of childhood illness

	Burundi	DRC	Nigeria	Pooled Sample
Malaria	*N* = 144	*N* = 289	*N* = 419	*N* = 852
Gave antimalarial (artemisinin-based or quinine)	135 (93.8%)	243 (84.1%)	361 (86.2%)	739 (86.7%)
*Pneumonia or other respiratory infection*	*N* = 144	*N* = 289	*N* = 0	*N* = 433
Gave any antibiotic	117 (81.3%)	207 (71.6%)	-	324 (74.8%)
*Dehydration*	*N* = 0	*N* = 289	*N* = 417	*N* = 706
Gave fluids (ORS, ringer’s lactate, or other saline solution)	-	274 (94.8%)	268 (64.3%)	542 (76.8%)

Dashes indicate items for which data were unavailable because relevant vignettes were not asked in all countries

The observed adherence to clinical guidelines was significantly lower than the average provider knowledge (Table 3). In the pooled sample, 76% of patients diagnosed with malaria were prescribed antimalarial medication, ranging from 52% in Nigeria to 96% in Burundi. 74% of patients diagnosed with pneumonia or another respiratory infection were given antibiotics (40% in Nigeria, 81% in the DRC, and 95% in Burundi), and only 51% of patients diagnosed with dehydration were given ORS or IV fluids (27% in Nigeria and 61% in the DRC). For respiratory infections and malaria, adherence was substantially lower when using the stricter definitions of treatment adherence: 31% of patients diagnosed with pneumonia or other severe respiratory infection were given amoxicillin (11% in Nigeria and 37% in the DRC), and 60% of patients diagnosed with malaria were given artemisinin-containing antimalarial medications (50% in Nigeria, 56% in the DRC, and 92% in Burundi).

**Table 3:** Technical quality of treatment provided

	Burundi	DRC	Nigeria	Pooled Sample
	*n (%)*	*n (%)*	*n (%)*	*n (%)*
*Patients diagnosed with malaria* ^*1*^	*N* = 131	*N* = 612	*N* = 183	*N* = 926
Gave any antimalarial drug (artemisinin or quinine)	126 (96.2%)	481 (78.6%)	95 (51.9%)	702 (75.8%)
Gave artemisinin-containing antimalarial	120 (91.6%)	340 (55.6%)	92 (50.3%)	552 (59.6%)
*Patients diagnosed with pneumonia or other respiratory infection* ^*2*^	*N* = 20	*N* = 181	*N* = 55	*N* = 256
Gave any antibiotic	19 (95.0%)	146 (80.7%)	22 (40.0%)	199 (73.7%)
Gave amoxicillin^3^	-	67 (37.0%)	6 (10.9%)	73 (30.9%)
*Patients diagnosed with dehydration* ^*4*^	*N* = 0	*N* = 69	*N* = 30	*N* = 99
Gave fluids (ORS, ringer’s lactate, or other saline solution)	-	42 (60.9%)	8 (26.7%)	50 (50.5%)

^1^The sample size for DRC 612 because treatment information is missing for 58 of the 670 participants in the DRC who were diagnosed with malaria

^2^Data not available in Burundi; pooled sample size is 236

^3^Treatment information was missing for 17 of the 181 children in the DRC who were diagnosed with pneumonia or other respiratory infection

^4^Treatment information was missing for the 6 patients in Burundi diagnosed with dehydration. Treatment information was missing for 3 of the 72 patients in the DRC diagnosed with dehydration

**Fig. 1:**
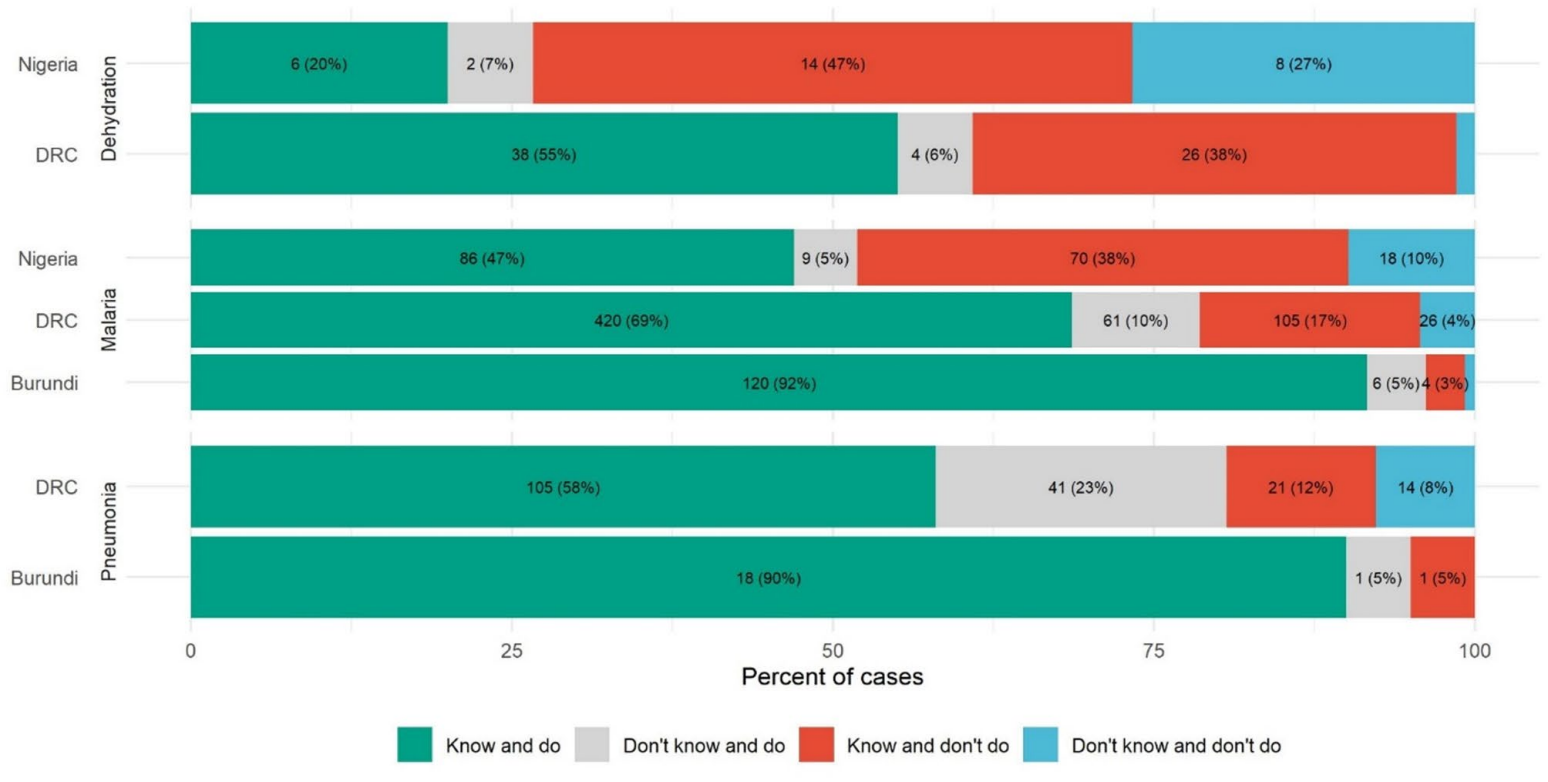
Know-do gaps in care for pneumonia, malaria, and dehydration. Notes: Horizontal bars represent the full set of patients diagnosed with a given condition (dehydration, malaria, or pneumonia or other severe respiratory infectoin) in each country (Nigeria, the Democratic Republic of the Congo (DRC), or Burundi). Green shading indicates the subset of cases in which the provider responded correctly to the relevant clinical vignette, and the patient received the recommended treatment for their diagnosed condition (“know and do”). Gray shading indicates the subset of cases in which the provider responded incorrectly to the relevant clinical vignette, and the patient received the recommended treatment for their diagnosed condition (“don’t know and do”). Red shading indicates the subset of cases in which the provider responded correctly to the relevant vignette but did not give the patient the recommended treatment (“know and don’t do”); this is the “know-do gap.” Blue shading indicates the subset of cases in which the provider responded incorrectly to the relevant vignette and did not give the patient the recommended treatment (“don’t know and don’t do”). Together, the green and gray bars represent the total percentage of patients who received the correct treatment. Together, the red and blue bars represent the adherence gap

Across all country-condition pairs, lack of knowledge explained less than half of the non-adherence to clinical guidelines (Fig. 1). For example, in the DRC, 79% of patients diagnosed with malaria received antimalarial medication (combining the “know and do” bar with the “don’t know and do” bar). However, among the 22% of patients who did not receive antimalarial medication (“don’t know and don’t do” and “know and don’t do”), more than three quarters were treated by providers who responded to the malaria vignette case correctly. Know-do gaps varied widely, and were largest for dehydration treatment in Nigeria and smallest for malaria treatment in Burundi. We also observed a substantial share of cases (from 5% to 23% across country-disease pairs) in which providers treated the patient correctly even though they responded incorrectly to the corresponding vignette (“don’t know and do”). The results were similar when we restricted the sample to health facilities with confirmed stock of the relevant medication (Tables S5-S6), and when we restricted to cases that were diagnosed as severe (Table S7).

Our regression analysis identified several factors that were associated with correct treatment in the DRC, conditional on provider knowledge (Table 4). Patients seen by older providers were significantly more likely to receive correct treatment. Patients in health centers were 10% points (pps) (95% CI: 0–21) more likely to receive correct treatment than patients in hospitals. None of the factors included in the analysis were significantly associated with correct treatment in Burundi or Nigeria. Bivariate analyses showed similar patterns (Tables S8-S12).

**Table 4:** Associations with correct treatment, conditional on knowing the correct treatment

	(1)	(2)	(3)
	Burundi	DRC	Nigeria
Facility type (Ref = Hospital)			
Reference health center		0.037	
		(-0.100 - 0.174)	
Health center		0.104*	-0.138
		(-0.002 - 0.210)	(-1.039 - 0.764)
Health post			-0.319
			(-1.409 - 0.770)
Cadre (Ref = Other)			
Doctor		0.020	0.062
		(-0.213 - 0.253)	(-0.607 - 0.732)
Nurse	-0.027	-0.031	0.076
	(-0.098 - 0.044)	(-0.228 - 0.166)	(-0.369 - 0.520)
Community health worker			0.037
			(-0.370 - 0.443)
In-service IMCI training		0.042	-0.017
		(-0.045 - 0.128)	(-0.182 - 0.147)
Provider age (Ref = 18-29)			
Age 30-39	-0.045	0.182***	0.152
	(-0.118 - 0.028)	(0.072 - 0.292)	(-0.323 - 0.628)
Age 40-49	-0.025	0.140**	0.275
	(-0.113 - 0.063)	(0.026 - 0.254)	(-0.192 - 0.743)
Age 50+	-0.005	0.190***	0.161
	(-0.232 - 0.223)	(0.060 - 0.321)	(-0.326 - 0.648)
Constant	1.005***	0.570***	0.438
	(0.928 - 1.081)	(0.332 - 0.808)	(-0.539 - 1.414)
Observations	140	557	163
R-squared	0.014	0.0352	0.025
ci in parentheses			

Figure shows results from linear regression of a binary indicator for correct treatment of all relevant conditions (1 if the patient was correctly treated for all conditions with which he or she was diagnosed) on health facility and provider characteristics. The sample is restricted to cases in which patients were seen by providers who correctly answered the relevant knowledge vignette(s) corresponding to the conditions with which the patient was diagnosed. Data on in-service IMCI training were not collected in Burundi

***p<0.01, **p<0.05, *p<0.1

## Discussion

In this study in Burundi, the DRC, and Nigeria, we compared the technical quality of care for sick children to health care provider knowledge to understand potential reasons for low technical quality of care. In our sample, knowledge of treatment guidelines was good on average, with over 75% of providers familiar with essential guidelines for malaria, respiratory infections, and dehydration. However, many providers knew the recommendations in the guidelines did not follow them in practice. We found that closing know-do gaps – i.e., all providers applying their knowledge in practice – could improve guideline adherence by between 38 and 47 pps for dehydration, 3–38 pps for malaria, and 5–12 pps for pneumonia and other severe respiratory infections.

Our findings build on a growing literature that documents know-do gaps in a wide range of geographic settings and clinical domains [14–19, 37, 38], including child health care. For example, one study in Ethiopia found that providers scored 39 pps higher on knowledge assessments for malaria care than on assessments of the technical quality of care they provided [17]. Even when patients were diagnosed with malaria, only 30% were given artesunate or quinine [17]. In rural India, 72% of providers reported that they would offer ORS to children with dehydration, but only 17% actually provided it when visited by standardized patients with the relevant symptoms [16]. Our study expands this evidence base with results from three additional countries. We found higher technical quality of care than the earlier studies, but still substantial know-do gaps.

We found significant underuse of antibiotics among patients diagnosed with pneumonia or other respiratory infections in the DRC and Nigeria. This finding is particularly striking in light of evidence of the very high rates of antibiotic use in many countries in sub-Saharan Africa [39]. This could suggest that antibiotic use is not only high but also poorly targeted. Providers’ failure to prescribe antimalarials to patients diagnosed with malaria (particularly in Nigeria) is also very puzzling.

We did not find any significant variation in know-do gaps by provider cadre, consistent with past evidence [40]. Surprisingly, in the DRC, we found that technical quality of care was lower and know-do gaps were larger in hospitals than in health centers. A past study found similar technical quality of care for child dehydration in health facilities and hospitals in the DRC, so it seems that hospitals do not necessarily provide higher-quality basic child health care in this setting [41]. In the DRC, we also found smaller know-do gaps among older providers, suggesting that performance may improve with experience.

Of the conditions studied, we observed the lowest technical quality of care for dehydration, with only 52% of patients receiving ORS or IV fluids, and know-do gaps of 38% points in the DRC and 47% points in Nigeria. In most dehydration cases (84%), the provider recommended that the caregiver give their child more fluids to drink, but this is not sufficient according to the guidelines. One explanation could be that providers do not judge these cases as serious enough to warrant ORS or IV fluids. However, even among the 33 patients in the sample who were diagnosed with severe dehydration, only 12 (36%) were treated with ORS or IV fluids. Underuse of ORS is a longstanding challenge in many countries [42], and there is a clear need to understand why providers so often fail to follow basic guidelines for dehydration treatment in the study settings. A recent study in Bihar and Karnataka, India points to a possible explanation: misalignment between patient preferences for ORS and providers’ perceptions of those preferences [43]. In survey data there, the majority of caregivers expressed a preference for ORS, but providers believed that most caregivers would not want ORS. In a randomized experiment, researchers found that the rate of ORS prescribing was 27% points higher when standardized patients expressed a preference for ORS compared with when they expressed no preference; provider misperceptions of patient preferences explained 42% of the gap in ORS prescriptions [43].

We observed a significant number of cases (ranging from 5 to 23% across country-condition pairs) in which providers gave the correct treatment even though they did not answer the corresponding vignette correctly. We see several possible explanations for these cases of “don’t know and do.” First, they could signal a limitation in the use of clinical vignettes to measure knowledge; some providers may, for example, be better at recognizing an illness case in person than when the symptoms are described to them. Second, it is possible that providers give certain treatments (such as antibiotics) even if they do not believe they are necessary; in this case, they might not mention it in a knowledge assessment but might give it in practice. Thirdly, it is possible that, in some cases, there was more than one provider present during a consultation, and the additional provider(s) influenced the treatment decisions. Finally, in these cases, accompanying caregivers may have asked for the right medication during consultations.

This study raises the important question of how to address know-do gaps. Our findings are aligned with past studies showing limited impacts of in-service training interventions [44, 45]. The literature points to several potential ways forward. Firstly, at the level of the health system, there may be a need for management interventions that focus on improving health care quality; past studies have highlighted the potential benefits of investments in district health management teams [46] or in facility-level management and governance [47]. At the level of health care providers, past studies suggest that incentives, whether monetary [48] or non-monetary [38, 49, 50], can affect providers’ behaviors and adherence to clinical guidelines. Performance-based financing interventions, which provide payments to facilities and providers, have been effective in improving the quality of care in some settings, though not all [32, 51]. Finally, recent research with standardized patients suggests that patient behavior during health consultations – e.g., whether patients clearly communicate their symptoms [52] or express preferences for correct treatments [43] – can shape the quality of care they receive. There is a need for further research to identify real-world interventions that can improve health care quality by leveraging patient demand.

This study has several limitations. First, variations in study protocols across countries meant that the results were not directly comparable in all cases. We therefore focus on individual country findings without making direct comparisons in country performance. Still, we draw qualitatively similar conclusions across countries; the adherence gaps and know-do gaps we estimate are large enough that small differences in methodologies are unlikely to matter for our main conclusions. Future studies could aim to strengthen cross-country comparisons using standardized data collection approaches such as in the World Bank’s Service Delivery Indicator surveys [53]. Second, quality measurement was based on direct observation of care which can be affected by Hawthorne effects [54]. If present, Hawthorne effects would bias our results in favor of higher quality care but would not necessarily change the cross- or within-country comparisons. Third, our determination of patients’ diagnoses is based on the diagnoses of observed providers; a potentially better approach may have been to have a qualified provider reevaluate each case to determine the diagnosis. Our approach is likely to again bias our adherence estimates upwards, given evidence of low adherence to IMCI guidelines for diagnosis of childhood illnesses [55]. Our know-do gap findings are even more striking since our assessment of knowledge (which required providers to diagnose imaginary patients) was in some ways stricter than our assessment of quality (which assumed providers’ diagnoses were correct). Finally, this analysis is based on data collected from 2013 to 2015; quality of care in these settings may have improved since these data were collected.

## Conclusion

In conclusion, we documented extremely low adherence to guidelines for the management of common childhood illnesses even among providers who are aware of them. This study suggests that interventions to improve technical quality of care need to go beyond addressing gaps in provider knowledge, to also ensure that providers apply their knowledge in clinical practice.

## Supplementary Information

Supplementary Material 1.

## Data Availability

All data and analytic code are available upon request from the study authors.

## References

[1] World Bank. World Bank Databank. Available from: https://data.worldbank.org/indicator. Accessed 2 Oct 2025. Cited 14 Aug 2025.

[2] Institute for Health Metrics and Evaluation. GBD Compare. Available from: https://vizhub.healthdata.org/gbd-compare/. Cited 12 Dec 2018.

[3] Uwemedimo OT, Lewis TP, Essien EA, Chan GJ, Nsona H, Kruk ME, et al. Distribution and determinants of pneumonia diagnosis using integrated management of childhood illness guidelines: a nationally representative study in Malawi. BMJ Global Health. 2018;3(2):e000506. 29662688 10.1136/bmjgh-2017-000506 PMC5898357

[4] Kruk ME, Gage AD, Mbaruku GM, Leslie HH. Content of Care in 15,000 Sick Child Consultations in Nine Lower-Income Countries. Health Services Research. Available from: 10.1111/1475-6773.12842. Cited 29 June 2018. 10.1111/1475-6773.12842 PMC6052007 29516468

[5] Lunze K, Biemba G, Lawrence JJ, MacLeod WB, Yeboah-Antwi K, Musokotwane K, et al. Clinical management of children with fever: a cross-sectional study of quality of care in rural Zambia. Bull World Health Organ. 2017;95(5):333–42. 28479634 10.2471/BLT.16.170092 PMC5418822

[6] Clarke-Deelder E, Shapira G, Samaha H, Fritsche GB, Fink G. Quality of care for children with severe disease in the Democratic Republic of the congo. BMC Public Health. 2019;19(1):1608. 31791291 10.1186/s12889-019-7853-3 PMC6889659

[7] Krüger C, Heinzel-Gutenbrunner M, Ali M. Adherence to the integrated management of childhood illness guidelines in Namibia, Kenya, Tanzania and Uganda: evidence from the national service provision assessment surveys. BMC Health Services Research . 2017;17(1). Available from: https://bmchealthservres.biomedcentral.com/articles/10.1186/s12913-017-2781-3. Cited 17 Dec 2017. 10.1186/s12913-017-2781-3 PMC5729502 29237494

[8] Lange S, Mwisongo A, Mæstad O. Why don’t clinicians adhere more consistently to guidelines for the Integrated Management of Childhood Illness (IMCI)? Soc Sci Med. 2014;104:56–63. 24581062 10.1016/j.socscimed.2013.12.020

[9] Reñosa MD, Dalglish S, Bärnighausen K, McMahon S. Key challenges of health care workers in implementing the integrated management of childhood illnesses (IMCI) program: a scoping review. Global Health Action. 2020;13(1):1732669. 32114968 10.1080/16549716.2020.1732669 PMC7067189

[10] Kruk ME, Gage AD, Arsenault C, Jordan K, Leslie HH, Roder-DeWan S, et al. High-quality health systems in the sustainable development goals era: time for a revolution. Lancet Global Health. 2018;6(11):e1196–252. 30196093 10.1016/S2214-109X(18)30386-3 PMC7734391

[11] United Nations General Assembly. Transforming our world: the 2030 Agenda for Sustainable Development. 2015 Oct. Report No.: A/Res/70/1. Available from: https://www

[12] Leonard KL, Masatu MC. The use of direct clinician observation and vignettes for health services quality evaluation in developing countries. Soc Sci Med. 2005;61(9):1944–51. 15936863 10.1016/j.socscimed.2005.03.043

[13] Rokicki S, Mwesigwa B, Cohen JL. Know-do gaps in obstetric and newborn care quality in uganda: a cross‐sectional study in rural health facilities. Trop Med Int Health. 2021;26(5):535–45. 33529436 10.1111/tmi.13557

[14] Boffa J, Moyo S, Chikovore J, Salomon A, Daniels B, Kwan AT, et al. Quality of care for tuberculosis and HIV in the private health sector: a cross-sectional, standardised patient study in South Africa. BMJ Glob Health. 2021;6(5):e005250. 33990360 10.1136/bmjgh-2021-005250 PMC8127976

[15] Das J, Kwan A, Daniels B, Satyanarayana S, Subbaraman R, Bergkvist S, et al. Use of standardised patients to assess quality of tuberculosis care: a pilot, cross-sectional study. Lancet Infect Dis. 2015;15(11):1305–13. 26268690 10.1016/S1473-3099(15)00077-8 PMC4633317

[16] Mohanan M, Vera-Hernández M, Das V, Giardili S, Goldhaber-Fiebert JD, Rabin TL, et al. The Know-Do gap in quality of health care for childhood diarrhea and pneumonia in rural India. JAMA Pediatr. 2015;169(4):349. 25686357 10.1001/jamapediatrics.2014.3445 PMC5023324

[17] Gage AD, Kruk ME, Girma T, Lemango ET. The know-do gap in sick child care in Ethiopia. PLoS ONE. 2018;13(12):e0208898. 10.1371/journal.pone.0208898 PMC6291134 30540855

[18] Ray Saraswati L, Baker M, Mishra A, Bhandari P, Rai A, Mishra P, et al. Know-Can gap: gap between knowledge and skills related to childhood diarrhoea and pneumonia among frontline workers in rural Uttar Pradesh, India. Trop Med Int Health. 2020;25(4):454–66. 31863613 10.1111/tmi.13365

[19] Ibnat F, Leonard K, Bawo L, Mohammed-Roberts R. The Three-Gap Model of Health Worker Performance. Washington, DC: The World Bank; (Policy Research Working Paper). Report No.: No. 8782. Available from: https://openknowledge.worldbank.org/handle/10986/31408. Accessed 2 Oct 2025.

[20] Gove S. Integrated management of childhood illness by outpatient health workers: technical basis and overview. The WHO working group on guidelines for integrated management of the sick child. Bull World Health Organ. 1997;75(Suppl 1):7. PMC2486995 9529714

[21] World Health Organization. Child Health and Development. Integrated management of childhood illness. Available from: https://www.who.int/teams/maternal-newborn-child-adolescent-health-and-ageing/child-health/integrated-management-of-childhood-illness. Cited 15 Aug 2025.

[22] World Health Organization. Integrated Management of Childhood Illness global survey report. 2017. Available from: http://apps.who.int/iris/handle/10665/258963. Cited 27 Nov 2018.

[23] Nimpagaritse M, Korachais C, Nsengiyumva G, Macq J, Meessen B. Addressing malnutrition among children in routine care: how is the integrated management of childhood illnesses strategy implemented at health centre level in burundi? BMC Nutr. 2019;5(1):22. 32153935 10.1186/s40795-019-0282-y PMC7050905

[24] Boschi-Pinto C, Labadie G, Dilip TR, Oliphant N, Dalglish SL, Aboubaker S, et al. Global implementation survey of integrated management of childhood illness (IMCI): 20 years on. BMJ Open. 2018;8(7):e019079. 10.1136/bmjopen-2017-019079 PMC6067364 30061428

[25] Ogah PO, Uguru N, Okeke C, Mohammed N, Ogbe O, Ashiver WG, et al. Primary health care in nigeria: best practices and quality of care in Nigeria. BMC Health Serv Res. 2024;24(1):963. 39169323 10.1186/s12913-024-11406-0 PMC11337854

[26] McLaughlin M, Metiboba L, Giwa A, Femi-Ojo O, Ravi N, Mahmoud NM, et al. Adherence to integrated management of childhood illness (IMCI) guidelines by community health workers in Kano State, Nigeria through use of a clinical decision support (CDS) platform. BMC Health Serv Res. 2024;24(1):953. 39164647 10.1186/s12913-024-11245-z PMC11337650

[27] Maini R, Hotchkiss DR, Borghi J. A cross-sectional study of the income sources of primary care health workers in the Democratic Republic of congo. Hum Resour Health. 2017;15(1):17. 28219445 10.1186/s12960-017-0185-4 PMC5322790

[28] World Bank Group. Performance-based Financing in the Health Sector of the Democratic Republic of Congo: Impact Evaluation Report. World Bank. 2022. Available from: http://elibrary.worldbank.org/doi/book/10.1596/38132. Cited 28 May 2024.

[29] World Bank Group. Impact Evaluation of Nigeria State Health Investment Project. 2018. Available from: https://documents1.worldbank.org/curated/en/589301552969360031/pdf/NSHIP-IE-Report.pdf. Accessed 2 Oct 2025.

[30] Korachais C, Nkurunziza S, Nimpagaritse M, Meessen B. Impact of the extension of a performance-based financing scheme to nutrition services in Burundi on malnutrition prevention and management among children below five: A cluster-randomized control trial. Adu-Afarwuah S, editor. PLoS One. 2020;15(19):e0239036. 32946500 10.1371/journal.pone.0239036 PMC7500612

[31] Khanna M, Loevinsohn B, Pradhan E, Fadeyibi O, McGee K, Odutolu O, et al. Decentralized facility financing versus performance-based payments in primary health care: a large-scale randomized controlled trial in Nigeria. BMC Med. 2021;19(1):224. 34544415 10.1186/s12916-021-02092-4 PMC8452448

[32] Shapira G, Clarke-Deelder E, Booto BM, Samaha H, Fritsche GB, Muvudi M, et al. Impacts of performance-based financing on health system performance: evidence from the Democratic Republic of congo. BMC Med. 2023;21(1):381. 37794389 10.1186/s12916-023-03062-8 PMC10552286

[33] Shapira G, Fink G. The World Bank Microdata Library. Health Results Based Financing Impact Evaluation 2015. Available from: https://microdata.worldbank.org/index.php/catalog/2825/data-dictionaryn. Accessed 2 Oct 2025.

[34] Kandpal E. State Health Investment Project: Impact Evaluation Endline Survey, 2017. Available from: https://microdata.worldbank.org/index.php/catalog/4042. Cited 24 Jul 2024.

[35] Nimpagaritse M, Korachais C, Roberfroid D, Kolsteren P, Zine Eddine El Idrissi MD, Meessen B. Measuring and Understanding the effects of a performance based financing scheme applied to nutrition services in Burundi—a mixed method impact evaluation design. Int J Equity Health. 2016;15(1):93. 27301741 10.1186/s12939-016-0382-0 PMC4908705

[36] Mack C, Su Z, Westreich D. Types of Missing Data. In: Managing Missing Data in Patient Registries: Addendum to Registries for Evaluating Patient Outcomes: A User’s Guide. Third Edition. Rockville, Maryland, USA: Agency for Healthcare Research and Quality (US); 2018. Available from: https://www 29671990

[37] Jackson D, Shahabuddin ASM, Sharkey AB, Källander K, Muñiz M, Mwamba R, et al. Closing the know-do gap for child health: unicef’s experiences from embedding implementation research in child health and nutrition programming. Implement Sci Commun. 2021;2(1):112. 34588002 10.1186/s43058-021-00207-9 PMC8479889

[38] Leonard KL, Masatu MC. Professionalism and the know-do gap: exploring intrinsic motivation among health workers in Tanzania. Health Econ. 2010;19(12):1461–77. 19960481 10.1002/hec.1564

[39] Fink G, D’Acremont V, Leslie HH, Cohen J. Antibiotic exposure among children younger than 5 years in low-income and middle-income countries: a cross-sectional study of nationally representative facility-based and household-based surveys. Lancet Infect Dis. 2020;20(2):179–87. 31843383 10.1016/S1473-3099(19)30572-9

[40] Das J, Hammer J, Leonard K. The quality of medical advice in Low-Income countries. J Economic Perspect. 2008;22(2):28. 10.1257/jep.22.2.93 19768841

[41] Arsenault C, Kim MK, Aryal A, Faye A, Joseph JP, Kassa M, et al. Hospital-provision of essential primary care in 56 countries: determinants and quality. Bull World Health Organ. 2020;98(11):735–D746. 33177770 10.2471/BLT.19.245563 PMC7607473

[42] Sreeramareddy CT, Low YP, Forsberg BC. Slow progress in diarrhea case management in low and middle income countries: evidence from cross-sectional national surveys, 1985–2012. BMC Pediatrics. 2017;17(1). Available from: http://bmcpediatr.biomedcentral.com/articles/10.1186/s12887-017-0836-6. Cited 31 Oct 2018. 10.1186/s12887-017-0836-6 PMC5360044 28320354

[43] Wagner Z, Mohanan M, Zutshi R, Mukherji A, Sood N. What drives poor quality of care for child diarrhea? Experimental evidence from India. Science. 2024;383(6683):eadj9986. 38330118 10.1126/science.adj9986 PMC12057796

[44] Rowe AK, Rowe SY, Peters DH, Holloway KA, Chalker J, Ross-Degnan D. Effectiveness of strategies to improve health-care provider practices in low-income and middle-income countries: a systematic review. Lancet Global Health. 2018;6(11):e1163–75. 30309799 10.1016/S2214-109X(18)30398-X PMC6185992

[45] Leslie HH, Gage A, Nsona H, Hirschhorn LR, Kruk ME. Training and supervision did not meaningfully improve quality of care for pregnant women or sick children in Sub-Saharan Africa. Health Aff. 2016 Sept;35(9):1716–24. 10.1377/hlthaff.2016.0261 27605655

[46] Doherty T, Tran N, Sanders D, Dalglish SL, Hipgrave D, Rasanathan K et al. Role of district health management teams in child health strategies. BMJ. 2018; k2823. 10.1136/bmj.k2823 PMC6063257 30061110

[47] Lewis TP, McConnell M, Aryal A, Irimu G, Mehata S, Mrisho M, et al. Health service quality in 2929 facilities in six low-income and middle-income countries: a positive deviance analysis. Lancet Global Health. 2023;11(6):e862-70. 37202022 10.1016/S2214-109X(23)00163-8 PMC10205971

[48] Das J, Holla A, Mohpal A, Muralidharan K. Quality and accountability in health care delivery: audit-study evidence from primary care in India. Am Econ Rev. 2016;106(12):3765–99. 29553219 10.1257/aer.20151138

[49] Brock M, Lange A, Leonard KL. Generosity norms and intrinsic motivation in health care provision: evidence from the laboratory and the field.:27.

[50] Leonard KL, Masatu MC, Vialou A. Getting doctors to do their best: the roles of ability and motivation in health care quality. J Hum Resour. 2007;42(3):682–700.

[51] De Walque D, Kandpal E. Reviewing the evidence on health financing for effective coverage: do financial incentives work? BMJ Glob Health. 2022;7(9):e009932. 10.1136/bmjgh-2022-009932 PMC9490608 36130774

[52] Kovacs RJ, Lagarde M, Cairns J. Can patients improve the quality of care they receive? Experimental evidence from Senegal. World Dev. 2022;150:105740. 35115735 10.1016/j.worlddev.2021.105740 PMC8651629

[53] The World Bank. Service Delivery Indicators: Health. Available from: https://www.worldbank.org/en/programs/service-delivery-indicators/health. Cited 28 May 2024.

[54] Leonard K, Masatu MC. Outpatient process quality evaluation and the Hawthorne effect. Soc Sci Med. 2006;63(9):2330–40. 16887245 10.1016/j.socscimed.2006.06.003

[55] Perales NA, Wei D, Khadka A, Leslie HH, Hamadou S, Yama GC, et al. Quality of clinical assessment and child mortality: a three-country cross-sectional study. Health Policy Plann. 2020;35(7):878–87. 10.1093/heapol/czaa048 32577749

