# Supplementary material for: Know-do gaps in the clinical management of childhood illness: evidence from three countries in sub-Saharan Africa
Source: BMC Public Health. 2025 Oct 24;25:3601. doi: 10.1186/s12889-025-24852-0 (PMC12553165; doi:10.1186/s12889-025-24852-0)
Supplement: Supplementary file 1 — Supplementary Material 1. [file 12889_2025_24852_MOESM1_ESM.docx]

**Supplementary Appendix to “Know-do gaps in the clinical management of childhood illness: evidence from three countries in sub-Saharan Africa”**

**Table S1**: Comparison of malaria vignettes

| **MALARIA** | | | | |
| --- | --- | --- | --- | --- |
| *Country*  *(survey round, vignette #)* | *Patient characteristics and symptoms described in vignette* | *Correct diagnosis per IMCI guidelines* | *Correct treatment per vignette answer key* | *Vignette implementation* |
| DRC  (Case 5) | - Girl aged 16 months, normal height and weight - Diarrhea - Temperature 38.5 - Difficulty waking - Cries a lot - Cold - Normal respiration - No convulsions - Throat and ears are normal - No cough, neck is normal - Eats normally - No signs of dehydration | - Malaria - Diarrhea (not severe dehydration) | - Test for malaria - Prescribe ORS for treatment at home - Give a dose of paracetamol - Prescribe an antimalarial (Artesunate Lumefantrine, AA) | **Treatment options prompted**: The case was fully described to the provider. Then, the treatment options were read to the provider. The provider was asked to agree or disagree with each treatment option. |
| Nigeria  (Case 2) | - Boy aged 7 months - Fever coming and going for 24 hours - Already had paracetamol and one dose of choloroquine from a neighbor; vomited afterwards - No danger signs - Assessment suggests malaria - No RDTs or microscopy in the facility | - Malaria | - Treat for malaria with ACT - Advise patient to return to PHC immediately if the condition worsens or develop danger signs or any adverse drug reaction is noticed | **Treatment options NOT prompted:** The case was fully described to the provider. The treatment options were not read to the provider. The provider was asked to come up with the correct treatment without being prompted. |
| Burundi (Case 3) | - Girl aged 18 months, weight 7.3 kg - No breastfeeding problems, no problems with appetite - Diarrhea sometimes, but not currently - Sometimes cramps - Fever for the last two days, some sweating and chills - MUAC Of 120 mm - No rash - Malaria test positive - Temperature 37.8, pulse 110, breathing 28 per minute - No dehydration | - Malaria (not severe) | - Prescribe recommended first-line antimalarial - Explain danger signs to the mother | **Dynamic case**: The provider accessed information about the case by asking questions. The treatment options were not read to the provider. The provider was asked to come up with the correct treatment without being prompted. |

**Table S2**: Comparison of pneumonia or other respiratory infection vignettes

| **PNEUMONIA OR OTHER RESPIRATORY INFECTION** | | | | |
| --- | --- | --- | --- | --- |
| *Country*  *(survey round, vignette #)* | *Patient characteristics and symptoms described in vignette* | *Correct diagnosis per IMCI guidelines* | *Correct treatment per vignette answer key* | *Vignette implementation* |
| DRC (Case 3) | - Girl aged 13 months, weight 8.5 kg and temperature 38.8 - Cough for five days and fever last night - Generalized rash 1 month ago - Mother continues to breastfeed - No malaria in the area - Stridor and sunken chest - Vaccination card is complete, received vitamin A 4 months ago | - Severe pneumonia | - Refer to a hospital - Give a dose of antibiotic - Advise the mother to continue breastfeeding | **Treatment options prompted**: The case was fully described to the provider. Then, the treatment options were read to the provider. The provider was asked to agree or disagree with each treatment option. |
| Burundi  (Case 1) | - 13-month-old boy, 6.6 kg and 69.8 c, MUAC = 115mm - Falls asleep when trying to breastfeed - No vomiting, no convulsions - Seems tired all the time - No diarrhea now, but a few times in the past 2 months - Cough for 5 days, sometimes dry and sometimes productive - Breathing a bit faster than usual - Decreased appetite; Since yesterday, only had a bit of rice one time (normally would eat 3 times per day and breastfeed 2-3 times per day) - Fever: temperature 38.6 - Pulse 130 per minute - Breathing 52 per minute - Sunken chest - Pale tongue, no problems with ears - Runny nose - Gave paracetamol two days ago - Feet not swollen - No rash - Negative malaria test - Hemoglobin 9.1 | - Severe pneumonia (because of lethargy and cough) | - Give first dose of an appropriate antibiotic - Refer urgently to the hospital | **Dynamic case**: The provider accessed information about the case by asking questions. The treatment options were not read to the provider. The provider was asked to come up with the correct treatment without being prompted. |

**Table S3**: Comparison of diarrhea or dehydration vignettes

| **DIARRHEA OR DEHYDRATION** | | | | |
| --- | --- | --- | --- | --- |
| *Country*  *(survey round, vignette #)* | *Patient characteristics and symptoms described in vignette* | *Correct diagnosis per IMCI guidelines* | *Correct treatment per vignette answer key* | *Vignette implementation* |
| DRC  (Case 1) | - Girl aged 12 months, weight 9kg - Diarrhea for the past 2 days - Liquid and mucosal stool - Diarrhea immediately after eating - Vomits at every meal - No fever, no convulsions - No tears when she cries - Lethargic - Skin pinch goes back very slowly - No cholera at the moment | - Severe dehydration | - Refer to hospital - Administer ringer’s lactate or saline solution by IV - Administer a dose of injectable antibiotic - Give a dose of vitamin A - Give a dose of zinc - Counsel to continue breastfeeding | **Treatment options prompted**: The case was fully described to the provider. Then, the treatment options were read to the provider. The provider was asked to agree or disagree with each treatment option. |
| Nigeria  (Case 4) | - Girl, aged 25 months and weight 10.5kg - Asleep since morning and could not wake up - Has not eaten or drank anything since yesterday - No vomiting, no convulsions - Diarrhea for six days - Runny nose - Lethargic - Skin pinch returned slowly - No other abnormal signs - No cholera in the area now (*this was omitted from the Nigeria baseline case) | - Severe dehydration | - Recommend urgent referral to a hospital - Advise on giving ORS on the way to hospital   OR   - Administer ringer lactate or normal saline IV solution - Administer ORS at the facility - (If cholera in area: give antibiotic for cholera) – only relevant for Nigeria baseline case | **Treatment options NOT prompted:** The case was fully described to the provider. The treatment options were not read to the provider. The provider was asked to come up with the correct treatment without being prompted. |

**Figure S1**: Sample flowchart

**
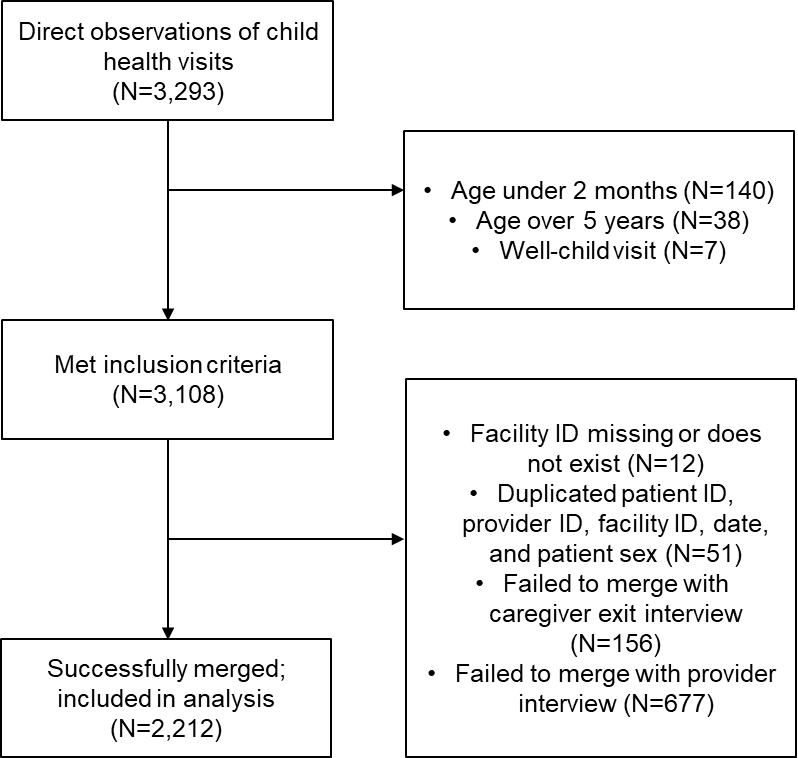
**

**Table S4**: Provider knowledge, restricted to providers observed caring for the relevant cases

|  | **Burundi** | **DRC** | **Nigeria** | **Pooled Sample** |
| --- | --- | --- | --- | --- |
| ***Malaria*** | **N=79** | **N=264** | **N=137** | **N=480** |
| Gave antimalarial (artemisinin-based or quinine) | 75 (94.9%) | 225 (85.2%) | 118 (86.1%) | 418 (87.1%) |
| ***Pneumonia or other respiratory infection*** | **N=17** | **N=122** | **N=0** | **N=141** |
| Gave any antibiotic | 16 (94.1%) | 86 (70.5%) | - | 104 (73.8%) |
| ***Dehydration*** | **N=0** | **N=56** | **N=30** | **N=86** |
| Gave fluids (ORS or ringer’s lactate) | - | 54 (96.4%) | 20 (66.7%) | 74 (86.1%) |

**Table S5**: Stock-outs

|  | DRC | Nigeria |
| --- | --- | --- |
|  | N=159* | N=379* |
| Amoxicillin pills: none in stock | 32.1% | 56.2% |
| Amoxicillin syrup: none in stock | 43.4% | 60.9% |
| Artesunate + amodiaquine: none in stock | 42.1% |  |
| Artesunate + lumefantrine: none in stock | 86.8% |  |
| Fansidar: none in stock | 37.1% | 71.2% |
| ORS: none in stock | 27.0% | 59.1% |

Note: Data on supply was missing from 96 facilities in the DRC. It was not collected in Burundi.

**Table S6**: Know-do gaps, conditional on having available stock (pooled sample)

|  | Antibiotics for pneumonia (N=91) | Antimalarials for malaria (N=338) | Fluids for dehydration (N=39) |
| --- | --- | --- | --- |
| Don’t know and don’t do | 6 (6%) | 19 (6%) | 3 (8%) |
| Know and don’t do | 11 (12%) | 66 (20%) | 17 (44%) |
| Don’t know and do | 25 (27%) | 19 (6%) | 0 (0%) |
| Know and do | 49 (53%) | 234 (69%) | 19 (48%) |

**Table S7**: Know-do gaps in severe cases of pneumonia and dehydration (pooled sample)

|  | Antibiotics for severe pneumonia (N=91) | Fluids for severe dehydration (N=33) |
| --- | --- | --- |
| Don’t know and don’t do | 3 (18%) | 7 (21%) |
| Know and don’t do | 1 (6%) | 14 (42%) |
| Don’t know and do | 3 (18%) | 2 (6%) |
| Know and do | 10 (59%) | 10 (30%) |

**Table S8**: Know-do gaps by facility type and condition (DRC)

|  | Total | Hospital | Health Center of Reference | Health Center / CSI(CAM) | p-value |
| --- | --- | --- | --- | --- | --- |
| *Know-do gap: all diagnosed conditions* | *N=701* | *N=123* | *N=66* | *N=512* | 0.14 |
| Knowledge gap | 7.0% | 6.5% | 9.1% | 6.8% |  |
| Know-do gap | 17.5% | 24.4% | 21.2% | 15.4% |  |
| Correct treatment | 75.5% | 69.1% | 69.7% | 77.7% |  |
| *Know-do gap: any antibiotic for pneumonia cases* | *N=180* | *N=30* | *N=15* | *N=135* | <0.001 |
| Knowledge gap | 7.7% | 0.0% | 0.0% | 10.3% |  |
| Know-do gap | 11.6% | 30.0% | 33.3% | 5.1% |  |
| Correct treatment | 80.7% | 70.0% | 66.7% | 84.6% |  |
| *Know-do gap: any antimalarial for malaria cases* | *N=613* | *N=108* | *N=58* | *N=447* | 0.018 |
| Knowledge gap | 4.2% | 6.5% | 10.3% | 2.9% |  |
| Know-do gap | 17.2% | 21.3% | 20.7% | 15.7% |  |
| Correct treatment | 78.6% | 72.2% | 69.0% | 81.4% |  |
| *Know-do gap: any fluids for dehydration cases* | *N=69* | *N=18* | *N=4* | *N=47* | 0.94 |
| Knowledge gap | 1% | 0% | 0% | 2% |  |
| Know-do gap | 38% | 39% | 25% | 38% |  |
| Correct treatment | 61% | 61% | 75% | 60% |  |

**Table S9**: Know-do gaps by facility type and condition (Nigeria)

|  | Total | Hospital | Health Center / CSI(CAM) | Health Post | p-value |
| --- | --- | --- | --- | --- | --- |
| *Know-do gap: all diagnosed conditions* | N=198 | N=2 | N=193 | N=3 | *0.85* |
| Knowledge gap | 12.1% | 0.0% | 12.4% | 0.0% |  |
| Know-do gap | 38.9% | 50.0% | 38.3% | 66.7% |  |
| Correct treatment | 49.0% | 50.0% | 49.2% | 33.3% |  |
| *Know-do gap: any antimalarial for malaria cases* | N=183 | N=2 | N=178 | N=3 | 0.86 |
| Knowledge gap | 9.8% | 0.0% | 10.1% | 0.0% |  |
| Know-do gap | 38.3% | 50.0% | 37.6% | 66.7% |  |
| Correct treatment | 51.9% | 50.0% | 52.2% | 33.3% |  |
| *Know-do gap: any fluids for dehydration cases* | N=30 | 0 | N=30 | 0 | *-* |
| Knowledge gap | 27% |  | 27% |  |  |
| Know-do gap | 47% |  | 47% |  |  |
| Correct treatment | 27% |  | 27% |  |  |

**Table S10**: Know-do gaps by cadre (Burundi)

|  | Total | Nurse | Other | p-value |
| --- | --- | --- | --- | --- |
| *Know-do gap: all diagnosed conditions* | *N=148* | *N=97* | *N=51* | *0.31* |
| Knowledge gap | 0.7% | 0.0% | 2.0% |  |
| Know-do gap | 3.4% | 4.1% | 2.0% |  |
| Correct treatment | 95.9% | 95.9% | 96.1% |  |
| *Know-do gap: antimalarial (any) for malaria cases* | *N=131* | *N=87* | *N=44* | *0.35* |
| Knowledge gap | 0.8% | 0.0% | 2.3% |  |
| Know-do gap | 3.1% | 3.4% | 2.3% |  |
| Correct treatment | 96.2% | 96.6% | 95.5% |  |
| *Know-do gap: any antibiotic for pneumonia cases* | *N=20* | *N=13* | *N=7* | *0.45* |
| Knowledge gap | 0% | 0% | 0% |  |
| Know-do gap | 5% | 8% | 0% |  |
| Correct treatment | 95% | 92% | 100% |  |

**Table S11**: Know-do gaps by cadre (DRC)

|  | Total | Doctor | Nurse | Other | p-value |
| --- | --- | --- | --- | --- | --- |
| *Know-do gap: all diagnosed conditions* | *N=701* | *N=78* | *N=598* | *N=25* | 0.64 |
| Knowledge gap | 7.0% | 3.8% | 7.5% | 4.0% |  |
| Know-do gap | 17.5% | 19.2% | 17.6% | 12.0% |  |
| Correct treatment | 75.5% | 76.9% | 74.9% | 84.0% |  |
| *Know-do gap: any antibiotic for pneumonia cases* | *N=181* | *N=13* | *N=157* | *N=11* | 0.36 |
| Knowledge gap | 7.7% | 7.7% | 8.3% | 0.0% |  |
| Know-do gap | 11.6% | 23.1% | 11.5% | 0.0% |  |
| Correct treatment | 80.7% | 69.2% | 80.3% | 100.0% |  |
| *Know-do gap: any antimalarial for malaria cases* | *N=612* | *N=69* | *N=526* | *N=17* | 0.81 |
| Knowledge gap | 4.2% | 1.4% | 4.6% | 5.9% |  |
| Know-do gap | 17.2% | 17.4% | 17.1% | 17.6% |  |
| Correct treatment | 78.6% | 81.2% | 78.3% | 76.5% |  |
| *Know-do gap: any fluids for dehydration cases* | *N=69* | *N=14* | *N=54* | *N=1* | 0.91 |
| Knowledge gap | 1% | 0% | 2% | 0% |  |
| Know-do gap | 38% | 36% | 39% | 0% |  |
| Correct treatment | 61% | 64% | 59% | 100% |  |

**Table S12**: Know-do gaps by cadre (Nigeria)

|  | Total | Doctor | Nurse | CHW | Other | p-value |
| --- | --- | --- | --- | --- | --- | --- |
| *Know-do gap: all diagnosed conditions* | *N=197* | *N=7* | *N=28* | *N=151* | *N=11* | 0.81 |
| Knowledge gap | 12.2% | 14.3% | 7.1% | 12.6% | 18.2% |  |
| Know-do gap | 39.1% | 42.9% | 39.3% | 39.1% | 36.4% |  |
| Correct treatment | 48.7% | 42.9% | 53.6% | 48.3% | 45.5% |  |
| *Know-do gap: any antimalarial for malaria cases* | *N=182* | *N=7* | *N=25* | *N=142* | *N=8* | 0.82 |
| Knowledge gap | 9.9% | 0.0% | 8.0% | 10.6% | 12.5% |  |
| Know-do gap | 38.5% | 42.9% | 36.0% | 38.7% | 37.5% |  |
| Correct treatment | 51.6% | 57.1% | 56.0% | 50.7% | 50.0% |  |
| *Know-do gap: any fluids for dehydration cases* | *N=30* | *N=1* | *N=3* | *N=23* | *N=3* | 0.67 |
| Knowledge gap | 27% | 100% | 0% | 26% | 33% |  |
| Know-do gap | 47% | 0% | 67% | 48% | 33% |  |
| Correct treatment | 27% | 0% | 33% | 26% | 33% |  |
